# Blunted responses to reward in remitted post-traumatic stress disorder

**DOI:** 10.1002/brb3.357

**Published:** 2015-06-11

**Authors:** Nilufer Kalebasi, Eveline Kuelen, Ulrich Schnyder, Sonja Schumacher, Christoph Mueller-Pfeiffer, Frank H Wilhelm, Jegath Athilingam, Hanspeter Moergeli, Chantal Martin-Soelch

**Affiliations:** 1 Department of Psychiatry and Psychotherapy, University Hospital Zurich, University of ZurichZurich, Switzerland; 2 Cantonal Hospital of TessinBellinzona, Switzerland; 3 Center of Education and Research (COEUR), Psychiatric Services of the County of St. Gallen-NorthWil, Switzerland; 4 Department of Psychiatry, Massachusetts General HospitalBoston, Massachusetts; 5 Division of Clinical Psychology, Psychotherapy and Health Psychology, University of SalzburgSalzburg, Austria; 6 Center for Integrative Neurosciences, University of California San FranciscoSan Francisco, California; 7 Division of Clinical and Health Psychology, Department of Psychology, University of FribourgFribourg, Switzerland

**Keywords:** Experimental, motivation, post-traumatic stress disorder, psychopathology, remitted, residual symptoms, reward

## Abstract

**Background:**

Recent evidence suggests blunted responses to rewarding stimuli in patients with post-traumatic stress disorder (PTSD). However, it is not clear whether these alterations in reward processing normalize in remitted PTSD patients.

**Methods:**

We tested behavioral and physiological responses to monetary reward in a spatial memory task in 13 accident survivors with remitted PTSD, 14 accident survivors who never had PTSD, and 16 nontrauma-exposed subjects. All accident survivors were recruited from two samples of severely physically injured patients, who had participated in previous prospective studies on the incidence of PTSD after accidental injury approximately 10 years ago. Reaction time, accuracy, skin conductance responses, and self-reported mood were assessed during the task.

**Results:**

Accident survivors who never had PTSD and nontrauma exposed controls reported significantly higher positive mood in the reinforced versus nonreinforced condition (*P *<* *0.045 and *P *<* *0.001, respectively), while there was no effect of reinforcement in remitted PTSD subjects.

**Conclusions:**

Our findings suggest an alteration of the reward system in remitted PTSD. Further research is needed to investigate whether altered reward processing is a residual characteristic in PTSD after remission of symptoms or, alternatively, a preexisting risk factor for the development of PTSD after a traumatic event.

## Introduction

Post-traumatic stress disorder (PTSD) can develop after exposure to actual or threatened death, serious injury, or sexual violence (American Psychiatric Association, 2013). Hallmark symptoms of PTSD are persistent reexperiencing of traumatic memories (e.g., in nightmares or flashbacks), persistent avoidance of stimuli reminiscent of the traumatic event (e.g., an avoidance of activities that arouse recollections of the trauma), and persistently increased autonomic arousal, which manifests in sleep disturbances, irritability, concentration problems, hypervigilance, and exaggerated startle responses.

Post-traumatic stress disorder has also been found to be associated with a reduced capacity for reward processing. Male Vietnam veterans with PTSD reported lower reward expectancies and lower satisfaction with received reward compared to male Vietnam veterans without PTSD when performing a wheel of fortune-type gambling task (Hopper et al. 2008). Using the same task, Elman et al. (2009) observed at a neural level lower activation in the striatum, a central region in the neural processing of reward information, to gains versus losses in PTSD patients compared to healthy controls. The reduced striatal activation to gains in PTSD patients was correlated at a behavioral level with self-reported motivational and social deficits. Moreover, in a rewarded decision-making task (Sailer et al. 2008), PTSD subjects were slower to learn the correct response pattern and; at the neural level, showed reduced activation in the nucleus accumbens and medial prefrontal cortex, two brain regions crucial for the neural processing of rewards (Knutson and Cooper 2005). Using the presentation of beautiful faces, a validated probe for natural rewards (Aharon et al., 2001), Elman et al. (2005) showed that male Vietnam veterans with PTSD spent less time viewing beautiful faces compared to male Vietnam veterans without PTSD, suggesting a reduced reaction to natural reward in PTSD. These blunted responses to rewards have been related to the symptoms of “emotional numbing” observed in PTSD that include diminished interest in significant activities, feelings of detachment from others, and restricted range of affect (Elman et al. 2005, American Psychiatric Association, 2013).

Despite ample evidence for a reduced capacity for reward processing in PTSD, it is not known whether this phenomenon is produced by the disorder or is rather independent of PTSD symptoms. In order to disentangle the basic dysfunction of the reward system from PTSD symptoms, we tested here whether there were alterations of the reward system in remitted PTSD patients.

We measured behavioral and physiological responses to monetary rewards in accident survivors with remitted PTSD, accident survivors who did not develop PTSD, and nontrauma-exposed healthy controls using a rewarded spatial memory task (Martin-Soelch et al. 2009). Since monetary wins have been shown to elicit increases in skin conductance levels (Wilkes et al. 2010), we measured skin conductance responses to monetary reward as well. We hypothesized that subjects with remitted PTSD would show reduced responses to reward as indicated by lower positive mood responses to monetary wins, a lack of a beneficial effect of reward on task performance (i.e., no decrease of reaction times and no higher accuracy), and lower skin conductance responses to reward, compared to trauma-exposed and nontrauma-exposed controls.

## Methods

### Participants

We tested 13 accident survivors with remitted PTSD (remitted PTSD), 14 accident survivors who had never qualified for a diagnosis of PTSD (trauma controls), and 16 healthy subjects without any traumatic experiences according to DSM-IV criteria (nontrauma controls), that defines trauma as the exposure to actual or threatened death or serious injury, or a threat to the physical integrity of self or others (American Psychiatric Association, 1994). The remitted PTSD subjects and trauma controls were recruited from two samples of severely physically injured patients (113 subjects with PTSD diagnosis and 343 without PTSD diagnosis) who had been hospitalized in the Department of Traumatology at the Zurich University Hospital approximately 10 years ago, and who had participated in previous prospective studies on the incidence of PTSD after accidental injury (Schnyder et al. 2001, 2008). In these studies, the presence of PTSD was assessed in the first month after the accident and again after 6, 12, and 36 months. The maximal CAPS scores obtained by the trauma controls and the remitted PTSD participants during the longitudinal studies following the accident are summarized in Table 1. Our initial intention to also include individuals with current PTSD from the same cohorts of accident survivors was abandoned because due a lack of suitable subjects (*N* = 3 subjects with current PTSD). Injuries experienced by the remitted PTSD and trauma controls were similar and included single or multiple bone fractures of the lower and /or upper limbs (for 10 trauma controls and seven remitted PTSD participants); fractures of cervical vertebrae (for three trauma controls and three remitted PTSD participants), artery’s injury (for one remitted PTSD participant), fractured skull (for one remitted PTSD participant), sternum fracture (for one trauma controls), and polytrauma (for one remitted PTSD). Primary inclusion criterion for the subjects with remitted PTSD and trauma controls was participation in the previous studies mentioned above. The remitted PTSD group must (1) have been diagnosed with full or subsyndromal PTSD according to DSM-IV criteria (American Psychiatric Association, 1994), as assessed by the Clinician-Administered PTSD Scale (CAPS, German version, Schnyder and Moergeli 2002) during the course of the longitudinal studies mentioned above, and (2) no longer satisfied PTSD criteria, assessed by the CAPS, at the time of this study. The trauma controls must have never fulfilled a diagnosis of full or subsyndromal PTSD following the accident during the previous longitudinal studies, or later as retrospectively assessed by the CAPS in this study. The healthy controls were recruited from the general population and had never experienced a traumatic event according to the DSM-IV trauma criterion (A1, A2) for PTSD, as assessed by the CAPS. Exclusion criteria for all subjects were a current diagnosis of PTSD, as assessed by the CAPS, or any other axis-I disorder as assessed by the Mini International Neuropsychiatric Interview (M.I.N.I.; Sheehan et al. 1998). All subjects were nonsmokers, right-handed, and without any psychoactive medication.

**Table 1:** Means and standard deviations for sociodemographic variables and clinical scores

	Trauma Controls (*N* = 14)		Remitted PTSD (*N* = 13)		Non-trauma Controls (*N* = 16)			
Gender (m/f)	6/8		5/8		6/10		χ^2^ = 0.9	
	Mean	SD	Mean	SD	Mean	SD	F/t	*P*
Age	58.64	7.10	54.00	10.12	54.06	10.31	1.15	0.33
Years of education	13.86	2.18	13.08	2.57	15.13	3.86	1.72	0.19
Verbal IQ	112.93	9.74	102.00	11.45	112.69	12.50	4.11	0.02^1^
Nonverbal IQ	82.86	12.45	87.08	13.79	95.94	15.37	3.43	0.04^2^
Spatial recall test part A	27.36	4.88	30.85	3.24	31.31	3.61	4.25	0.02^3^
Part B	3.86	1.51	4.00	1.73	5.06	1.57	2.56	0.09
Part A after B	5.21	2.23	5.92	1.19	4.94	2.24	0.93	0.41
Part A late recall	5.00	2.39	5.15	2.08	5.75	1.57	0.58	0.56
BDI	5.43	4.13	6.77	3.17	5.19	4.49	0.62	0.54
STAI- trait anxiety	31.71	4.58	33.92	4.54	35.50	6.60	1.83	0.17
PDS: total number of experienced traumas (lifetime)	1.57	1.45	1.85	1.46	—	—	0.49	0.63
PDS: number of traumatic events experienced before accident	0.36	0.63	0.62	0.87	—	—	0.89	0.38
PDS: number of traumatic events experienced after accident	1.21	1.19	1.23	1.24	—	—	0.04	0.97
CAPS total score	2.43	3.90	6.42	8.23	—	—	1.54	0.15
Maximal CAPS score after the accident	10.21	5.24	33.69	14.26	—	—	−5.60	<0.001
Years since accident	9.87	0.37	11.56	1.74	—	—	3.35	0.004
Years since remission			7.3	4.9				

PTSD, post-traumatic stress disorder; BDI, beck depression inventory; STAI, state-trait anxiety inventory; PDS, Post-traumatic stress diagnostic scale; CAPS, clinician-administered PTSD scale.

One-way ANOVAs (to compare all three groups) and independent samples *t*-tests (to compare trauma groups, only for PDS, and time since accident) were used to assess group differences. Post hoc tests, correcting for multiplicity according to Bonferroni (Bonferroni-corrected significance level of *P*: 0.5/3 comparisons = 0.016), were performed on variables showing significant differences in the ANOVAs to gauge specific differences between groups.

1 Post-hoc tests revealed no significant inter-group differences in verbal IQ: trauma controls versus remitted PTSD (*P* < 0.08), trauma controls versus non-trauma (*P* < 1.0), remitted PTSD versus non-trauma (*P* < 0.8).

2 Post-hoc tests revealed a significant difference (*P* < 0.03) between the trauma control and non-trauma groups in nonverbal IQ.

3 Post-hoc tests revealed a significant difference (*P* < 0.01) in spatial learning scores between the trauma control and non-trauma groups.

This study was approved by the local medical ethics committee. The participants were thoroughly informed about the study and gave written informed consent according to the Declaration of Helsinki.

### Psychometric assessments

The number of traumatic events experienced by the subjects was assessed with the German adaptation of the PDS (Post-traumatic Stress Diagnostic Scale; Foa 1995; German version, Ehlers et al. 1996). For remitted PTSD subjects and trauma controls, we specifically asked for number of traumatic events experienced before and after the accident. Intensity of depression and anxiety were measured with the Beck Depression Inventory (BDI; Beck et al. 1961; German version, Hautzinger et al. 1995) and the State-Trait Anxiety Inventory (STAI; Laux et al. 1981). We measured verbal intelligence using the “*Wortschatztest”* (WST; Metzler and Schmidt 1992), a multiple choice word comprehension test that is a German equivalent to the Spot-The-Word test (Baddeley et al. 1993). We used the *Wiener Matrizen-Test* (WMT; Formann and Piswanger 1979), a validated adaptation of the Raven’s Matrix test, as a measure of nonverbal intelligence. Both intelligence tests were presented as computer-based versions (Hogrefe Test System 2006). Memory and attentional functions were assessed using the Spatial Recall Test (7/24; Rao et al. 1984). The spatial recall test measures nonverbal learning function (part A), proactive interference (part B), retroactive interference (part A after B) and delayed recall.

### Experimental task

The subjects performed a spatial delayed response task adapted from Glahn et al. (2002), which was originally designed to investigate which brain regions responded to the systematic increase of cognitive load. This task was validated as a reward task in a previous study (Martin-Soelch et al. 2009), and the use of different levels of difficulty and different magnitude of rewards was able to differentiate between a subclinical sample (cannabis users and tobacco smokers) and a group of healthy participants. We therefore specifically chose this task as we expected the differences between the groups included in the present study to be subtle. The task was implemented, using E-Prime Version 2.0 Professional (Psychology Software Tools Inc., Pittsburgh, PA) and presented on a color monitor (Samsung, SyncMaster 191T, Gyeonggi-do, Korea) with a resolution of 1280 × 768 pixels.

**Figure 1:**
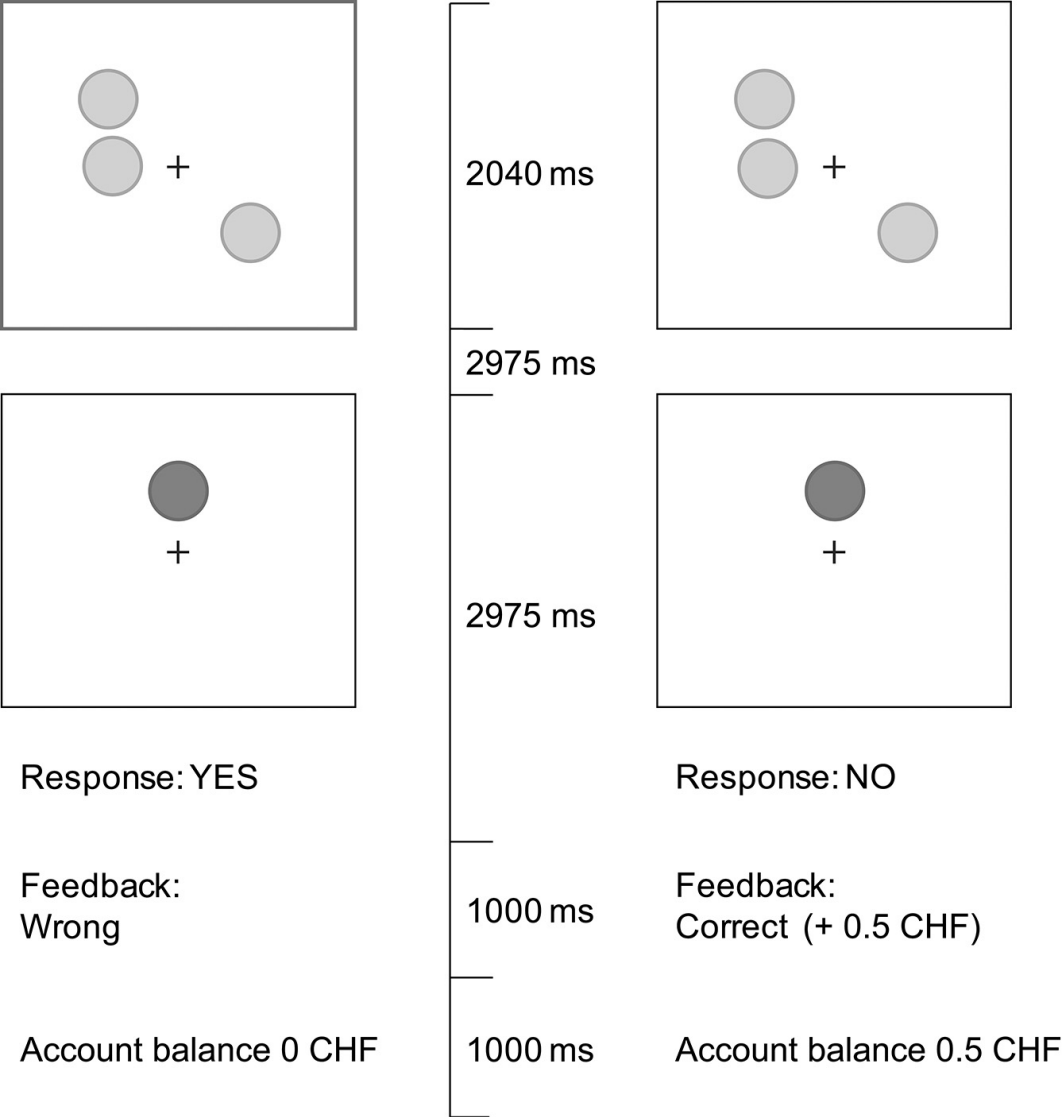
Illustration of a rewarded trial of the spatial delayed recall task at the easiest level of difficulty. In the first display, an array of yellow circles (3, 5 or 7) is presented for 2000 ms after a fixation time of 500 ms. After a delay of 3000 ms, a green circle appears and the subject has 3000 ms to decide whether the position of the green circle is the same as one of the preceding yellow circles by pressing two different buttons. After the subject has responded or the response time has elapsed, the circle disappears and the feedback and accumulated amount of earned money appears on the screen (in the rewarded condition) or the screen remains blank (in the unrewarded condition). During the rewarded condition, participants can earn a monetary reward for every correct response. The monetary reward increased according to the difficulty of the task. During the nonreinforced condition, participants eceive no feedback on their answer. The positions of the circles varied randomly and were organized according to a 5 × 5 grid dividing the space into 25 possible positions.

One trial of the task consisted of 3, 5, or 7 circles that were presented for 2040 ms, followed by a blank screen presented for 2975 ms, followed by a single circle presented until the subject responded (max. 2975 ms; Fig.1). Subjects had to identify whether the displayed circle appeared at the same spatial location as one of the previously shown circles and responded by pressing buttons on a computer keyboard. The task was presented in six blocks, comprising three levels of difficulty (3, 5 or 7 circles) and two reinforcement conditions (rewarded and unrewarded). Each block consisted of 12 trials with intertrial intervals of 510 ms, resulting in 72 trials across the entire task. Reaction time and accuracy were recorded for each trial.

During the rewarded condition, the participants earned a monetary reward for every correct response. The amount of the reward was related to the difficulty of the task, that is, CHF 0.50 (approximately USD 0.50) for three circles, CHF 1 for five circles, and CHF 2 for seven circles. After each rewarded trial, subjects received feedback regarding the accuracy of their response and the total amount of cash they won. The order of the block conditions was counterbalanced across subjects while the three rewarded blocks and the three unrewarded blocks were always grouped together. The maximum possible reward was CHF 42. Prior to the main experiment, subjects underwent a training block consisting of three trials from each difficulty level. Subjects rated their current mood from “very bad” to “very good” and their arousal from “not agitated” to “very agitated” on a 100-point visual analog scale (VAS) at the beginning of the experiment and after each block.

### Skin conductance measurement

Physiological data were acquired using a Biopac system MP150 (Biopac Systems, Inc, Goleta, CA). Skin conductance electrodes were placed on the thenar and hypothenar eminence of the left palmar surface, using Ag/AgCl electrodes filled with isotonic electrolyte gel. Skin conductance levels were sampled at 62.5 Hz.

## Data Analysis

### Behavioral data analysis

Statistical analyses were performed using PASW Statistics 18.0 (SPSS Inc., Chicago, IL). Linear mixed models analyses were performed on reaction time and accuracy for all trials with group (remitted PTSD, trauma controls, nontrauma controls), difficulty (3, 5, and seven circles), and reinforcement (rewarded, unrewarded) included as fixed effects and subject included as a random effect.

A one-way ANOVA was performed on baseline mood ratings in order to check for baseline mood differences across groups. Mood ratings from the six experimental blocks were then entered into a linear mixed models analysis with group, difficulty, and reinforcement included as fixed effects and subject included as a random effect.

For all mixed models analyses, the models were tested using all possible covariance types and optimized by the covariance type which produced the lowest Akaike’s Information Criterion (AIC). For reaction times the best fit was a first-order autoregressive moving average structure. For accuracy, the best fit was a scaled identity structure. For mood ratings, the best fit was a first-order autoregressive structure. Bonferroni corrected pairwise comparisons were used as post hoc tests for all mixed models analyses. Uncorrected pairwise comparisons were used to decompose effects which did not show up in the Bonferroni corrected post hoc tests. Level of significance was set at 0.05. Additional exploratory analyses tested for the effect of the groups’ differences in nonverbal memory and nonverbal IQ scores on accuracy and reaction times by introducing these scores as covariates, as they might have affected the performance at our nonverbal memory task.

The amount of money won was compared across groups using a one-way ANOVA.

### Physiological data analysis

Three subjects from the trauma control group were excluded from skin conductance analyses due to technical problems (resulting group size: *N *=* *11). Artifact correction and extraction of mean and maximum scores for condition and baseline intervals were performed with Autonomic Nervous System Laboratory 2.5 (ANSLAB; Wilhelm, F.H., & Peyk, P., 2005; available at the SPR Software Respository: http://www.sprweb.org). A skin conductance response for each condition was calculated as the difference between the highest level during the entire condition block and the mean level during a 2 sec interval immediately prior to the onset of the condition block. Skin conductance responses were log transformed (Ln(SCR + 1)) in order to normalize the distribution. Linear mixed models analyses were performed for all conditions with group, difficulty, and reinforcement included as fixed effects and subject included as a random effect. The best fit was obtained using a first-order autoregressive covariance structure. Bonferroni corrected pairwise comparisons were used as post hoc tests. Level of significance was set at 0.05.

## Results

### Subjects’ characteristics

Sociodemographic, psychometric, and clinical variables of the subjects are summarized by group in Table 1. The groups did not differ in age, sex, years of education, anxiety, and depression scores. Trauma controls achieved lower scores than nontrauma controls in the tests for verbal (*P *<* *0.03) and nonverbal intelligence (*P *<* *0.01) as well as for nonverbal learning (*P* < 0.02). Elapsed time since the accident was significantly longer (*P *<* *0.004) in the remitted PTSD subjects (M = 11.56, SD = 1.74) versus trauma controls (M = 9.87, SD = 0.37). Trauma-controls and remitted PTSD subjects differed significantly in the maximal CAPS scores that they had obtained after the accident (*P* < 0.001), but not in their current CAPS scores (*P* < 0.15). The averaged maximal CAPS scores obtained by the trauma controls and the remitted PTSD participants during the longitudinal studies following the accident are summarized in Table 1.”

### Behavioral results

Mean reaction times, mood scores, accuracy scores, and skin conductance responses by group, reinforcement condition, and the difficulty level are summarized in Table 1994.

**Table 2:** Estimated marginal means (M) and standard errors (SE) of the reaction times, mood ratings and accuracy for all levels of difficulty and reinforcement conditions of the rewarded spatial delayed recall task.

Difficulty	Reinforced						Non-reinforced					
	Level 1		Level 2		Level 3		Level 1		Level 2		Level 3	
	M	SE	M	SE	M	SE	M	SE	M	SE	M	SE
Reaction Time (ms)												
Non-Trauma Controls	1249.4	59.4	1331.4	59.2	1396.0	59.4	1288.1	59.4	1483.0	59.5	1436.4	59.6
Trauma Controls	1460.8	63.6	1495.4	63.3	1563.2	63.5	1466.2	63.5	1521.4	63.6	1692.3	63.8
Remitted PTSD	1309.5	66.0	1343.0	65.9	1419.1	65.6	1305.3	65.8	1424.3	66.0	1429.8	65.9
Mood Ratings (0: bad mood, 100: good mood)												
Non-Trauma Controls	79.1	5.3	80.0	5.3	77.3	5.3	72.2	5.3	68.7	5.3	65.8	5.3
Trauma Controls	83.1	5.6	73.5	5.6	78.8	5.6	74.9	5.6	73.5	5.6	71.9	5.6
Remitted PTSD	74.2	5.9	71.5	5.8	74.1	5.8	73.5	5.8	70.9	5.8	73.7	5.9
Accuracy (% correct responses)												
Non-Trauma Controls	79.2	3.6	72.4	3.6	60.9	3.6	84.9	3.6	71.9	3.6	65.6	3.6
Trauma Controls	75.6	3.9	58.3	3.9	54.8	3.9	75.6	3.9	63.1	3.9	51.2	3.9
Remitted PTSD	73.7	4.0	66.0	4.0	55.8	4.0	76.9	4.0	70.5	4.0	57.1	4.0
Skin conductance responses (*μ*Siemens)												
Non-trauma controls	1.36	0.32	1.42	0.32	1.10	0.32	0.90	0.32	1.36	0.32	0.99	0.32
Trauma controls	0.95	0.38	0.89	0.38	1.10	0.38	0.87	0.38	1.50	0.38	1.02	0.38
Remitted PTSD	0.94	0.37	0.65	0.37	1.77	0.37	0.91	0.37	1.73	0.37	1.26	0.37

PTSD, post-traumatic stress disorder.

The current mood was rated using a 100 mm visual analogue scale (0: bad mood, 100: good mood).

Levels of difficulty correspond to the number of circles to be remembered: level 1: 3 circles, level 2: 5 circles, and level 3: 7 circles. Accuracy is given in percent of correct responses.

#### Reaction time

**Figure 2:**
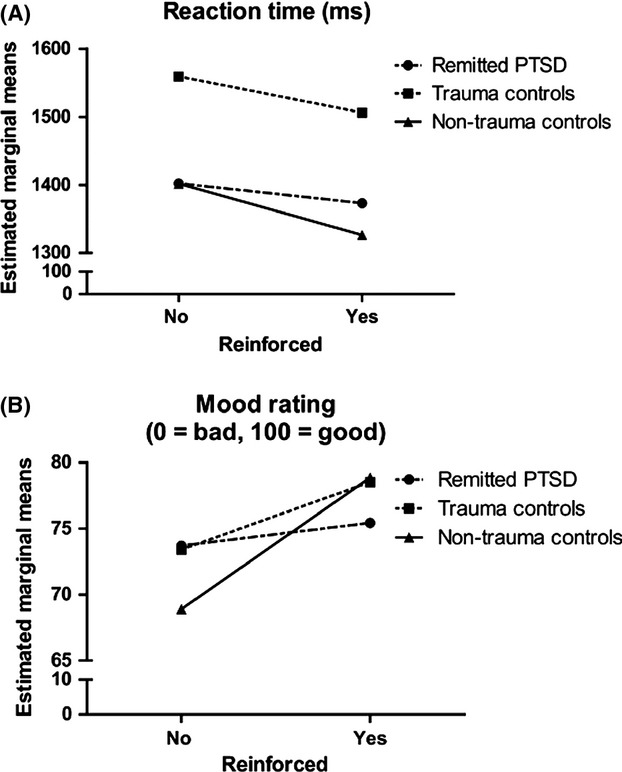
Plot of the reaction time and mood ratings across the different conditions of the task for each group of participants. (A) Reaction times: Across groups, the reaction times were slower with increasing difficulty and shorter for the reinforced condition. The nontrauma group showed the shortest reaction times and the longest reaction times were shown by the trauma controls. (B) Mood ratings: We found a significant group x reinforcement interaction (*F *=* *3.49, *P *=* *0.03) that is expressed by an effect of reinforcement in the trauma control (*P *=* *0.05) and nontrauma (*P *<* *0.001) groups but not for the remitted PTSD group (*P *=* *0.83).

We found significant main effects of group (*F *=* *3.31, *P *=* *0.047), difficulty (*F *=* *24.83, *P *<* *0.001), and reinforcement (*F *=* *5.65, *P *=* *0.019) on reaction time. Trauma-controls responded significantly slower than subjects with remitted PTSD and nontrauma controls (*P*s ≤ 0.04, Fig.2A). No significantly different reaction times were observed between remitted PTSD subjects and nontrauma controls (*P* = 0.9). Across groups, the reaction time was slower with increasing difficulty (three circles < five circles < seven circles; *P*s ≤ 0.02) and in the nonreinforced versus reinforced condition (*P* = 0.02) (Fig.2A). None of the interactions were significant (*P*’s ≥ 0.16). Adding the nonverbal IQ scores and the nonverbal learning scores as covariates did not affect the main effect of difficulty or reinforcement, but decreased the main effect of group (*P* < 0.1 resp. *P* < 0.09). However, none of these covariates was significant (nonverbal IQ: *P* < 0.48; nonverbal learning score: *P* < 0.69).

#### Accuracy

There was a statistical trend for a main effect of group (*F *=* *3.21; *P = *0.051) with lower accuracy in trauma controls versus nontrauma controls (*P = *0.05). Accuracy between other groups was comparable (*P*’s > 0.41). There was a significant main effect of difficulty (*F *=* *67.58; *P *<* *0.001) with significant deterioration of accuracy from low to intermediate to high difficulty levels (*P*’s < 0.001). Adding the nonverbal IQ scores and the nonverbal learning scores as covariates did not change the main effect of difficulty, but decreased the main effect of group (*P* < 0.29 resp. *P* < 0.13). The effect of nonverbal IQ, but not of nonverbal learning score, on accuracy was significant (*P* < 0.02 resp. *P* < 0.41).

#### Mood ratings

We found no group differences regarding mood ratings at baseline (*F *=* *0.75, *P *=* *0.48). Regarding mood rating scores during the task (Table 1994), we found significant main effects of difficulty (*F *=* *5.15, *P *=* *0.007) and reinforcement (*F *=* *12.65, *P *<* *0.001), and a significant group x reinforcement interaction (*F *=* *3.49, *P *=* *0.03). Decomposition of the interaction revealed that trauma controls and nontrauma controls reported significantly better mood in the reinforced versus nonreinforced condition (*P *<* *0.045 and *P *<* *0.001, respectively), while there was no effect of reinforcement in remitted PTSD subjects (Fig.2B).

#### Winnings

The nontrauma controls won on average CHF 28.1 (SD = 4.7), the trauma controls CHF 24.7 (SD = 4.6), and the remitted PTSD subjects CHF 25.7 (SD = 4.6). The total monetary rewards did not differ significantly between groups (*P *=* *0.13).

### Physiological results

There was a significant difficulty x reinforcement interaction (*P* = 0.02) on skin conductance responses. Decomposition of the interaction revealed that skin conductance responses were lower with versus without reinforcement in the intermediate difficulty level (*P* = 0.01). The only effect involving group was a statistical trend for the interaction of group x difficulty (*P* = 0.051). Remitted PTSD subjects showed a trend toward higher skin conductance responses than trauma controls in the high difficulty level (*P* = 0.051).

## Discussion

The aim of this study was to investigate the behavioral and physiological responses to monetary reward in remitted PTSD patients. In accordance with our hypothesis, we found a significant beneficial effect of reward on mood ratings in the trauma control group and the nontrauma control group, but not in the remitted PTSD group. This suggests an alteration in the responses to rewards in remitted PTSD.

Our observation of a lower beneficial effect of reward on mood ratings in remitted PTSD subjects extends previous findings of lower satisfaction with received reward (Hopper et al. 2008) and lower interest for motivational stimuli (Elman et al. 2005) in PTSD patients. Since we did not find any significant group differences in winnings, our results cannot be explained by differences in monetary gains. The dampened positive affective reaction to reward we observed in remitted PTSD subjects can neither be explained by a generally more negative mood in remitted PTSD subjects, because mood ratings at baseline were comparable between groups. Moreover, subjects in all groups reported similar levels of depression and anxiety, as measured by the BDI and STAI.

The lack of significant differences in task performance between remitted PTSD subjects and nontrauma controls in our study are in contrast with a previous study using a rewarded decision-making task, where PTSD subjects required more time to learn the correct response pattern (Sailer et al. 2008). As expected, we found faster reaction times in the reinforced versus nonreinforced condition across groups. Surprisingly, however, reaction times in trauma-controls were slower than in remitted PTSD subjects and nontrauma controls across conditions. Differences between trauma-resilient veterans and healthy controls in response to monetary rewards have previously been reported at the neural, but not at the behavioral level (Vythilingam et al. 2009), suggesting that the experience of a traumatic event can affect the reward system even without the subsequent development of PTSD. The slower reaction times in the trauma-controls compared to the nontrauma controls are, however, partly explained by the lower nonverbal intelligence scores and the poorer memory performance evidenced in this group, as revealed in the exploratory analyses using these scores as covariates. The observed faster reaction times during the rewarded trials confirm previous observations that monetary reward influences the qualitative aspects of performance, that is, the reaction times rather than the quantitative aspects of performance, that is, response accuracy, since we did not find any significant interaction between reward and accuracy (Martin-Soelch et al. 2009).

We did not observe any significant differences in skin conductance responses between groups. Subjects across groups showed lower skin conductance responses to rewarded versus nonrewarded trials in the middle level of difficulty. A trend toward higher skin conductance responses in the high difficulty level – independently of the reinforcement condition – was evidenced in the remitted PTSD (*P* = 0.051), suggesting a higher level of arousal with increased difficulty in the patients’ group. Previous studies investigating skin conductance responses to monetary reward in healthy subjects yielded inconsistent results. While one study reported higher skin conductance responses to monetary reward (Wilkes et al. 2010), other research groups reported higher skin conductance responses to monetary loss but not reward (Crone et al. 2004; Johnstone et al. 2007). The lack of group differences in skin conductance responses in our sample might also be explained by the methodological limitations discussed below.

The observation of group differences mainly at the self-reported level (i.e., the mood ratings) and not at the behavioral and / or physiological levels is in line with previous results obtained with our task, which observed group differences also only in the mood ratings (Martin-Soelch et al. 2009). Mood ratings associated with increasing rewards were also shown to be associated with the neural activation in the ventral striatum, one of the crucial regions for reward processing (Martin-Soelch et al. 2003).

Some limitations merit attention. The sample sizes were small, limiting our ability to find small group differences. We used a cross-sectional design for the testing of the reactions to reward and had no information about the subjects’ responses to reward prior to the accident or during the time they suffered from PTSD. With regard to the physiological responses to reward, we did not analyse SCR to reward per se, but quantified SCR as the peak score across a whole condition block as the timing of the experiment did not allow for event-related analyses. Keeping the increases of reward magnitude constant for the three levels of difficulty does not allow us to disentangle the relationship between task difficulty and reward magnitude. To address this issue, a latin-square design would have been used in future studies. Prospective studies are also needed to investigate whether alterations in the reward system in PTSD are a long-term consequence of PTSD or a pre-existing risk factor for the development of PTSD following a traumatic event. Furthermore, we did not include subjects with current PTSD, because we did not find a sufficient number of individuals from our previous cohorts of accident survivors who still suffered from PTSD.

In conclusion, we found a lower beneficial effect of reward on positive mood in accident survivors with remitted PTSD. This result suggests that the reward system is altered in remitted PTSD. Further research is needed to investigate whether an alteration in the reward system reflects a residual symptom of PTSD or, alternatively, a preexisting risk-factor for the development of PTSD in the aftermath of a traumatic event.

## References

[b502] Aharon I, Etcoff N, Ariely D, Chabris CF, O’Connor E, Breiter HC (2001). Beautiful faces have variable reward value: fMRI and behavioral evidence. Neuron.

[b2] American Psychiatric Association (1994). Diagnostic and statistical manual of mental disorders.

[b3] American Psychiatric Association (2013). Diagnostic and Statistical Manual of Mental Disorders.

[b4] Baddeley A, Emslie H, Nimmo-Smith I (1993). The Spot-the-Word test: a robust estimate of verbal intelligence based on lexical decision. Br. J. Clin. Psychol.

[b5] Beck AT, Ward CH, Mendelson M, Mock J, Erbaugh J (1961). An inventory for measuring depression. Arch. Gen. Psychiatry.

[b6] Crone EA, Somsen RJ, van Beek B, van der Molen MW (2004). Heart rate and skin conductance analysis of antecendents and consequences of decision making. Psychophysiology.

[b7] Ehlers A, Steil R, Winter H, Foa EB (1996). Deutsche Übersetzung der Posttraumatic Stress Diagnostic Scale (PDS).

[b8] Elman I, Ariely D, Mazar N, Aharon I, Lasko NB, Macklin ML (2005). Probing reward function in post-traumatic stress disorder with beautiful facial images. Psychiatry Res.

[b9] Elman I, Lowen S, Frederick BB, Chi W, Becerra L, Pitman RK (2009). Functional neuroimaging of reward circuitry responsivity to monetary gains and losses in posttraumatic stress disorder. Biol. Psychiatry.

[b10] Foa EB (1995). PDS (posttraumatic stress diagnostic scale): manual.

[b11] Formann A, Piswanger K (1979). Wiener Matrizen-Test (WMT): manual.

[b12] Glahn DC, Kim J, Cohen MS, Poutanen V-P, Therman S, Bava S (2002). Maintenance and manipulation of spatial working memory: dissociations in the prefrontal cortex. NeuroImage.

[b13] Hautzinger M, Bailer M, Worall H, Keller F (1995). Beck-Depressions-Inventar (BDI). Testhandbuch.

[b501] Hogrefe Test System [computer program] (2006).

[b14] Hopper JW, Pitman RK, Su Z, Heyman GM, Lasko NB, Macklin ML (2008). Probing reward function in posttraumatic stress disorder: expectancy and satisfaction with monetary gains and losses. J. Psychiatr. Res.

[b15] Johnstone T, van Reekum CM, Banziger T, Hird K, Kirsner K, Scherer KR (2007). The effects of difficulty and gain versus loss on vocal physiology and acoustics. Psychophysiology.

[b16] Knutson B, Cooper JC (2005). Functional magnetic resonance imaging of reward prediction. Curr. Opin. Neurol.

[b17] Laux L, Glanzmann P, Schaffner P, Spielberger CD (1981). Das State-Trait-Angstinventar.

[b18] Martin-Soelch C, Missimer J, Leenders K, Schultz W (2003). Neural activity related to the processing of increasing monetary reward in smokers and nonsmokers. Eur. J. Neurosci.

[b19] Martin-Soelch C, Kobel M, Stoecklin M, Michael T, Weber S, Krebs B (2009). Reduced response to reward in smokers and cannabis users. Neuropsychobiology.

[b20] Metzler P, Schmidt K-H (1992).

[b21] Rao S, Hammeke TA, Mcquillen MP, Kathri BO, Lloyd D (1984). Memory disturbance in chronic progressive multiple sclerosis. Arch. Neurol.

[b22] Sailer U, Robinson S, Fischmeister FP, Konig D, Oppenauer C, Lueger-Schuster B (2008). Altered reward processing in the nucleus accumbens and mesial prefrontal cortex of patients with posttraumatic stress disorder. Neuropsychologia.

[b23] Schnyder U, Moergeli H (2002). German version of clinician-administered PTSD scale. J. Trauma. Stress.

[b24] Schnyder U, Moergeli H, Klaghofer R, Buddeberg C (2001). Incidence and prediction of posttraumatic stress disorder symptoms in severely injured accident victims. Am. J. Psychiatry.

[b25] Schnyder U, Wittmann L, Friedrich-Perez J, Hepp U, Moergeli H (2008). Posttraumatic stress disorder following accidental injury: rule or exception in Switzerland?. Psychother. Psychosom.

[b26] Sheehan DV, Lecrubier Y, Sheehan KH, Amorim P, Janavs J, Weiller E (1998). The Mini-International Neuropsychiatric Interview (M.I.N.I.): the development and validation of a structured diagnostic psychiatric interview for DSM-IV and ICD-10. J. Clin. Psychiatry.

[b27] Vythilingam M, Nelson EE, Scaramozza M, Waldeck T, Hazlett G, Southwick SM (2009). Reward circuitry in resilience to severe trauma: an fMRI investigation of resilient special forces soldiers. Psychiatry Res.

[b503] Wilhelm FH, Peyk P (2005). http://www.sprweb.org.

[b28] Wilkes BL, Gonsalvez CJ, Blaszczynski A (2010). Capturing SCL and HR changes to win and loss events during gambling on electronic machines. Int. J. Psychophysiol.

